# Exceptional Specific Shielding Effectiveness of TOCNFs@MXene Hybrid Films via Densification Engineering

**DOI:** 10.3390/polym18080999

**Published:** 2026-04-20

**Authors:** Beibei Wang, Licheng Zhou, Sentao Wei, Jian Wang, Qun Wu, Chuan Cao, Kushairi Mohd Salleh

**Affiliations:** 1 Lab of Material Innovation Design and Intelligent Interaction, Zhejiang Sci-Tech University, Hangzhou 311199, China; ber@zstu.edu.cn (B.W.); 2025211205032@mails.zstu.edu.cn (L.Z.); 2024211205022@mails.zstu.edu.cn (S.W.); wangjian971212@163.com (J.W.); wuq@zstu.edu.cn (Q.W.); 2 School of Industrial Technology, Universiti Sains Malaysia, Penang 11800, Malaysia

**Keywords:** cellulose nanofibrils, MXene, hybrid films, electromagnetic interference shielding, mechanical properties

## Abstract

The rapid advancement of communication technologies exacerbates severe electromagnetic interference (EMI) pollution. Conventional flexible shielding materials rely heavily on non-degradable petroleum-based polymers, aggravating the electronic waste crisis. To address this dual challenge, sustainable biomass-derived TEMPO-oxidized cellulose nanofibrils (TOCNFs) emerge as ideal structural substrates. However, their intrinsic electrical insulation necessitates integrating conductive two-dimensional (2D) MXene, which suffers from severe self-restacking and brittleness. Herein, TOCNFs@MXene hybrid films are manufactured via vacuum filtration and hot-pressing densification. TOCNFs inhibit MXene self-restacking, constructing a highly ordered layered architecture via a dense hydrogen-bonded network. The optimized ultrathin film T5@M20 (~4.92 μm) exhibits an electrical conductivity of 1.09 × 10^6^ ± 5.06 × 10^4^ s m^−1^ and an X-band shielding effectiveness (SE_Total_) of 25.55 dB. Demonstrating an ultrahigh thickness-normalized specific shielding effectiveness (SSE/t) of 51,934.72 dB·cm^2^·g^−1^, this sustainable architecture shows exceptional potential for next-generation flexible electronics.

## 1. Introduction

With the rapid development of communication technologies and electronic devices, electromagnetic waves have permeated various sectors of daily life and industrial production with unprecedented penetration and intensity [1,2,3]. The widespread proliferation of this electromagnetic radiation has triggered severe EMI pollution, which not only significantly impairs the operational stability and data transmission security of precision electronic equipment but also poses potential threats to human health. Although traditional metal-based shielding materials exhibit excellent shielding effectiveness due to their superior electrical conductivity, their inherent drawbacks including high density, rigidity, susceptibility to corrosion, and processing difficulties, thus impeding their application in next-generation electronic devices, which demand lightweight, flexible, and highly integrated characteristics [4]. Currently, flexible electronics predominantly rely on non-degradable petroleum-based polymers as substrates, exacerbating the global e-waste crisis [5]. Therefore, the development of novel, high-performance EMI shielding materials that are simultaneously lightweight, flexible, mechanically robust, highly conductive, and environmentally friendly has emerged as a critical research imperative in the fields of materials science and electronic engineering.

Utilizing biomass materials for composite modification has been widely validated as an effective strategy [6,7,8,9]. TOCNFs, as a sustainable biomass-derived nanomaterial, stand out in this regard [10]. They possess a one-dimensional (1D) structure with a high aspect ratio analogous to carbon nanotubes, while the abundant oxygen-containing functional groups on their surface endow them with excellent water dispersibility and outstanding film-forming capabilities [11]. As a highly flexible, green, and mechanically robust building block, TOCNFs hold immense potential to serve as an eco-friendly structural substrate for next-generation flexible electronics, offering an ideal alternative to traditional synthetic polymers [12,13]. To impart superior electrical properties to the green biomass matrix, 2D transition metal carbides and MXene serve as an ideal functional filler, owing to their high electrical conductivity, excellent solution processability, and abundant surface functional groups [14,15]. However, constrained by strong interlayer van der Waals forces and hydrogen bonding, the film-forming process of MXene inevitably suffers from the self-restacking effect [16]. This induces stress concentration, causing the pure films to exhibit intrinsic brittleness, insufficient flexibility, and poor bending resistance. Consequently, achieving the efficient integration of the insulating biomass skeleton with the brittle 2D conductive nanosheets through rational micro/nano-structural design maximizing electrical performance while maintaining the flexibility of the composite system remains a pressing challenge in this field [17,18].

Despite the progress achieved in the TOCNFs and MXene component systems, there are still critical unresolved limitations in the existing literature. For instance, Zhan et al. constructed cellulose nanofiber and MXene hybrid films with enhanced mechanical properties, but mainly focused on the mechanical enhancement of the multi-level structure, and lacked a systematic and quantitative analysis of the regulation mechanism of densification engineering on the conductive network and electromagnetic shielding performance [19]. Our previous study [12] developed multifunctional MXene-based hybrid films, which adopted a multi-component composite system with a complex preparation process, making it difficult to accurately analyze the single-factor effect of densification on the structure and performance of the films. Meanwhile, research on ultrafine cellulose nanofiber and MXene hybrid films has paid more attention to the scalable manufacturing process, but did not reveal the intrinsic correlation between the densification degree, interfacial hydrogen bonding interaction and electromagnetic shielding mechanism [20]. In addition, most of the reported works inevitably introduced additional crosslinking agents, third-phase fillers or complex multi-step modification processes, which not only increased the complexity of preparation, but also brought uncontrollable interference to the analysis of the structure–activity relationship of the materials [21,22]. Therefore, it is still urgent to develop a facile binary system with simple preparation process, and to systematically reveal the densification mechanism and its regulation effect on EMI shielding performance, which is also the core starting point of this work.

**Figure 1:**
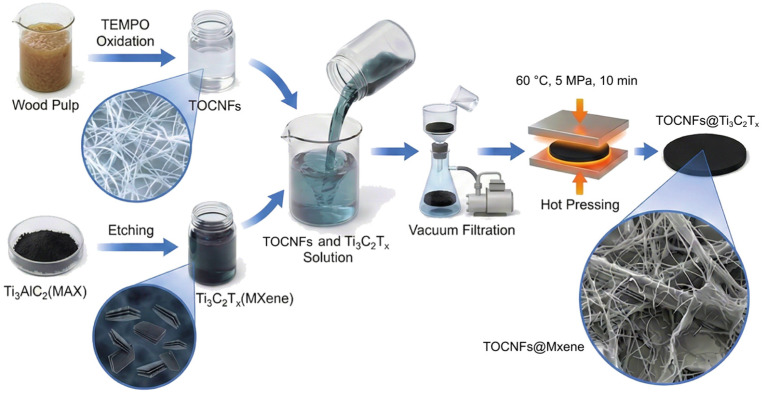
Schematic illustration for preparing TOCNFs@Ti_3_C_2_T*_x_* hybrid films. Original illustrations created via Rhino 8, Photoshop 2025, Figma, and digital drawing.

Herein, this study proposes a green and controllable strategy to successfully fabricate TOCNFs@Ti3C2T*_x_* hybrid films combining high flexibility, robust mechanical strength, and excellent electrical conductivity via the integration of vacuum filtration and hot-pressing densification. As illustrated in Figure 1, distinct from conventional natural drying processes, the hot-pressing densification technique not only effectively eliminates internal micro-void defects, thereby substantially reducing the contact resistance induced by the insulating TOCNFs, but also promotes the formation of stable interfacial hydrogen bonding interactions between the components. This innovative process successfully constructs a highly ordered layered network structure, endowing the as-prepared films with outstanding EMI shielding capabilities. Furthermore, benefiting from the intrinsic metallic conductivity of the MXene material and the mechanical stability imparted by the biomass substrate, this multifunctional integration technology paves a sustainable new pathway for the development of next-generation foldable electronic devices and wearable electronics.

## 2. Materials and Methods

### 2.1. Materials

Sodium bromide (NaBr, 99%), sodium hypochlorite (NaClO, 99%), sodium hydroxide (NaOH, 99%) and TEMPO were purchased from Sinopharm Chemical Reagent Co., Ltd. (Shanghai, China). Ti_3_AlC_2_ (MAX powder) was sourced from Zhengzhou Feynman Biotechnology Co., Ltd. (Zhengzhou, China). All reagents and chemicals were used as received without further purification.

### 2.2. Preparation of TOCNFs

TOCNFs were prepared via TEMPO-mediated oxidation. Briefly, softwood pulp was suspended in deionized water (DI, 100 mL, 1 wt.%) containing TEMPO (0.016 g, 0.1 × 10^−3^ M) and NaBr (0.1 g, 1 × 10^−3^ M). Oxidation was initiated by adding NaClO (5 mmol g^−1^) and maintained at pH 10.0–10.5 using 0.5 M NaOH. After reacting for 5 h, the process was quenched with ethanol and neutralized. The oxidized cellulose was washed, centrifuged, and finally homogenized to obtain a 1 wt.% TOCNFs dispersion.

### 2.3. Preparation of Ti_3_C_2_T_x_

Ti_3_C_2_T*_x_* was prepared by etching Ti_3_AlC_2_ (1.0 g) in a solution of LiF (1.6 g) and 9 M HCl (20 mL) at 45 °C for 24 h. Following repeated washing to neutral pH ≥ 6, the product was delaminated by ice-bath sonication and centrifuged at 3500 rpm to obtain a stable supernatant (5 mg mL^−1^).

### 2.4. Preparation of TOCNFs@Ti_3_C_2_T_x_ Hybrid Films

TOCNFs@Ti_3_C_2_T*_x_* hybrid films with varying mass ratios were fabricated via vacuum filtration coupled with hot-pressing densification. The as-prepared TOCNFs and Ti_3_C_2_T*_x_* dispersions were mixed according to predetermined mass ratios and diluted with DI water to yield a homogeneous suspension (20 mL) with a total concentration of 1.25 mg mL^−1^. Subsequently, the mixed suspension was homogenized via ultrasonication and deposited onto a filter membrane via vacuum filtration. To eliminate internal micro-voids and reinforce interfacial bonding, the resulting wet films, along with the filter membrane, were sandwiched between two smooth Teflon sheets and hot-pressed using a laboratory hydraulic press at 60 °C under a constant pressure of 5 MPa for 10 min. To systematically investigate the effect of component ratios, hybrid films were prepared with TOCNFs-to-Ti_3_C_2_T*_x_* mass ratios of 4:1, 3:2, 2:3, and 1:4. Pure TOCNFs (T25) and pure Ti_3_C_2_T*_x_* (M25) films were also fabricated as control groups, with the total mass of all samples maintained at 25 mg. The obtained hybrid films were denoted as Tx@My, where x and y represent the masses of TOCNFs and Ti_3_C_2_T*_x_*, respectively; for instance, T20@M5 refers to a film containing 20 mg of TOCNFs and 5 mg of Ti_3_C_2_T*_x_*.

### 2.5. Characterization

The morphology and microstructure of the films were observed using field emission scanning electron microscopy (FE-SEM, JSM-6700F, JEOL Ltd., Akishima, Japan) in SE mode and high-resolution transmission electron microscopy (HR-TEM, JEM-2100F, JEOL Ltd., Akishima, Japan). X-ray diffraction (XRD, SmartLab, Rigaku Corporation, Akishima, Japan) was conducted at 40 kV and 30 mA using Cu Kα1 radiation. Atomic force microscopy (AFM, Agilent 5500, Agilent Technologies Inc., Santa Clara, CA, USA) was employed in tapping mode to scan the TOCNFs and Ti_3_C_2_T*_x_* dispersions. The evolution of the crystal structure was characterized by Fourier transform infrared spectroscopy (FTIR, Thermo Scientific Nicolet iS5, Thermo Fisher Scientific Inc., Waltham, MA, USA), utilized to record the spectra of the films in the range of 400–4000 cm^−1^. Thermal stability was evaluated using a thermogravimetric analyzer (TGA, Netzsch STA 449 F5 Jupiter, NETZSCH-Gerätebau GmbH, Selb, Germany), from room temperature to 800 °C at a heating rate of 10 °C min^−1^.

Mechanical properties were tested using a microcomputer-controlled electronic universal material testing machine equipped with a 100 N load cell. The samples were cut into strips (9.5 × 25 mm^2^) and conditioned at room temperature (18 °C) and 25% relative humidity for at least 6 h prior to testing and the tensile rate was set to 0.5 mm min^−1^. At least five replicates were tested for each sample, and the results are expressed as average values (Zwick Z005). The thickness of the films was precisely measured using cross-sectional SEM images. The electrical conductivity of the strips was measured using a four-point probe tester (RTS-9, Guangzhou, China) by recording the sheet resistance, and the conductivity was calculated according to Equation (1).

(1) $$\sigma =\frac{1}{S}\cdot \frac{1}{R/L}=\frac{L}{R\cdot w\cdot t}$$

where *σ*, *R*, *L*, *S*, *w*, and *t* represent the electrical conductivity (S cm^−1^), sheet resistance (Ω sq^−1^), length (cm), cross-sectional area (cm^2^), width (cm), and thickness (cm), respectively. The electromagnetic interference shielding effectiveness (EMI SE) of the films (22.9 × 10.2 mm^2^) was measured within the X-band frequency range (8.2–12.4 GHz) using a vector network analyzer (VNA, N5244A PNA-X, Agilent Technologies, Inc., Santa Clara, CA, USA) via the coaxial method at room temperature. The EMI SE was calculated according to the Simon formula (Simon, 1981) [23]:

(2) $$SE=50+10log(\frac{\sigma }{f})+1.7t\sqrt{\sigma f}$$

The reflection coefficient (*R*), transmission coefficient (*T*), absorption coefficient (*A*), and total electromagnetic shielding effectiveness (*SE*) were calculated using the following equations:

(3) SE=SE+SE+SE

(4) $$R={\left|S_{11}\right|}^{2}={\left|S_{22}\right|}^{2}$$

(5) $$T={\left|S_{21}\right|}^{2}={\left|S_{12}\right|}^{2}$$

(6) A=1−R−T

The reflection loss (${SE}_{A}$), absorption loss (${SE}_{R}$), and total shielding effectiveness (${SE}_{T}$) (unit: dB) are calculated using the following equations:

(7) $${SE}_{R}=-10\log\left(1-R\right)$$

(8) $${SE}_{A}=-10\log\left(\frac{T}{1-R}\right)$$

(9) $${SE}_{T}={SE}_{R}+{SE}_{A}+{SE}_{M}$$

When ${SE}_{T}$ ≥ 15 dB, the multiple reflection loss ${SE}_{M}$ is generally considered negligible; therefore, ${SE}_{T}\approx {SE}_{R}+{SE}_{A}$. When taking the thickness and density of the material into account, the relevant equations are as follows:

(10) $$SSE=\frac{{SE}_{T}}{\rho }=dB\;{cm}^{3}{\;g}^{-1}$$

(11) $$SSE/t=\frac{SSE}{thickness}=dB\;{cm}^{2}{\;g}^{-1}$$

The formula for calculating EMI shielding efficiency (%) is:

(12) $$Shielding\;Efficiency\left(\%\right)=\left(1-10^{-\frac{{SE}_{T}}{m}}\right)\times 100\%$$

Regarding the preparation of this manuscript, Google Gemini 3.1 Pro was used for grammar checking and spelling correction and linguistic optimization to improve the readability of the text.

## 3. Results and Discussion

### 3.1. Morphology and Structure Characterization of TOCNFs@Ti_3_C_2_T_x_ Hybrid Films

**Figure 2:**
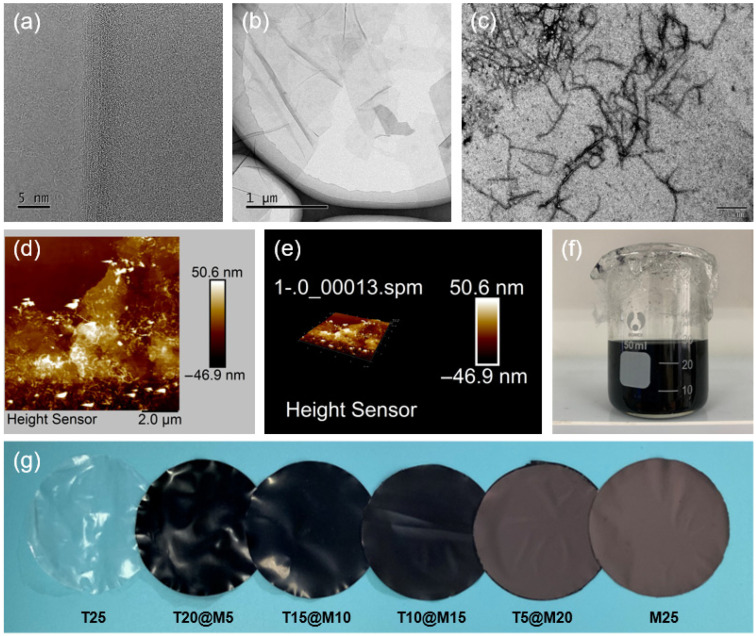
(**a**,**b**) TEM image of Ti_3_C_2_T*_x_* nanosheet; (**c**) TEM image of TOCNFs; (**d**,**e**) AFM images of TOCNFs; (**f**) TOCNFs@Ti_3_C_2_T*_x_* hybrid suspension; (**g**) TOCNFs@Ti_3_C_2_T*_x_* hybrid films with different mass ratios.

The micro-morphologies of the exfoliated Ti_3_C_2_T*_x_* nanosheets and TOCNFs were characterized using TEM and AFM. Figure 2a displays the TEM image of the edge of a Ti_3_C_2_T*_x_* nanosheet, clearly revealing its layered structure and ultrathin thickness. Figure 2b shows the TEM image of the Ti_3_C_2_T*_x_* nanosheets, which exhibit a 2D, ultrathin, transparent flake-like structure with a large lateral size. Their high transparency to the electron beam confirms their few-layer nature, indicating that the MAX phase was effectively exfoliated into few-layer or single-layer nanosheets. The TEM and AFM images of TOCNFs (Figure 2c–e) reveal a highly entangled, cobweb-like nanofibril network structure with a diameter of approximately 1–10 nm and a length of 100–500 nm, demonstrating an extremely high aspect ratio.

The stability of the hybrid dispersion and the macroscopic morphology of the films were subsequently evaluated. As shown in Figure 2f, the mixture of TOCNFs and Ti_3_C_2_T*_x_* formed a homogeneous and stable dark ink-like suspension without any noticeable precipitation or agglomeration after standing. This excellent compatibility is attributed to the abundant oxygen-containing functional groups on the surfaces of both TOCNFs and the hydrophilic Ti_3_C_2_T*_x_*, which facilitate the formation of strong interfacial hydrogen bonding [24,25]. Figure 2g presents optical photographs of the hybrid films with various mass ratios prepared via vacuum filtration. With the increase in Ti_3_C_2_T*_x_* content, the color of the films exhibited a distinct transition from the transparent plastic-like appearance of the T25 film to the characteristic dark gray with a metallic luster of the M25 film. The slight wrinkles observed on the film surface are likely caused by internal stress concentration or uneven shrinkage during the drying process.

**Figure 3:**
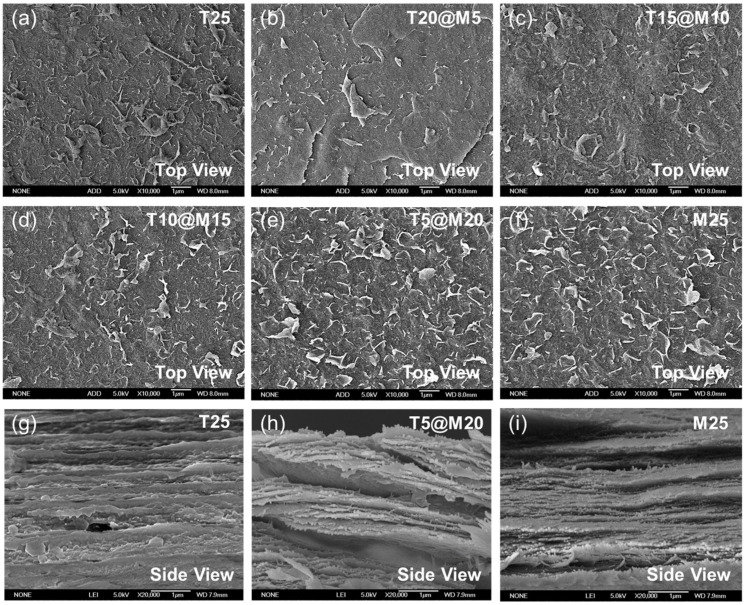
(**a**–**f**) SEM images of the T25, T20@M5, T15@M10, T10@M15, T5@M20 and M25 films; (**g**–**i**) Cross-sectional SEM images of T25, T5@M20 and M25 films.

SEM was employed to characterize the surface morphology of the films with different mass ratios (Figure 3a–i). Figure 3a,g presents the surface morphology of T25, which exhibits a dense and continuous surface with inherent micro-roughness resulting from the fibrous network formed during the vacuum filtration process. As shown in Figure 3b–e, the surface morphology of the hybrid films undergoes a significant change with the increase in the relative mass fraction of MXene. The MXene nanosheets are uniformly dispersed in the TOCNFs matrix without noticeable agglomeration, and the two components are uniformly intercalated to construct a typical nacre-inspired layered structure, where the 2D MXene nanosheets constitute the main skeleton of the layered structure, and the 1D TOCNFs form a continuous binding phase between the lamellae [26,27]. The sample shown in Figure 3e,h is T5@M20, where the high-content MXene nanosheets form a dense and continuous overlapping network on the surface, constructing an efficient pathway for electron transport. Despite the high MXene content, a small amount of TOCNFs can still serve as a nanobinder to fill the gaps between the nanosheets, eliminate interfacial defects, and maintain the dense structure of the film surface. During the hot-pressing process, the elevated temperature slightly softens the 1D TOCNFs polymer chains and enhances their mobility. Coupled with the applied vertical pressure, this enables the TOCNFs to effectively flow into and completely fill the micro-voids between the 2D MXene nanosheets, thereby yielding a highly aligned and densified nacre-like layered architecture [12]. Furthermore, the simultaneous application of heat and high pressure drives the functional groups on both MXene and TOCNFs into intimate proximity, thermodynamically facilitating the formation of a more robust intermolecular hydrogen-bonding network [28]. This one-step hot-pressing process also enables synchronous film densification and rapid drying to suppress MXene oxidation, and is characterized by low cost, facile operation and good scalability for industrial production.

**Figure 4:**
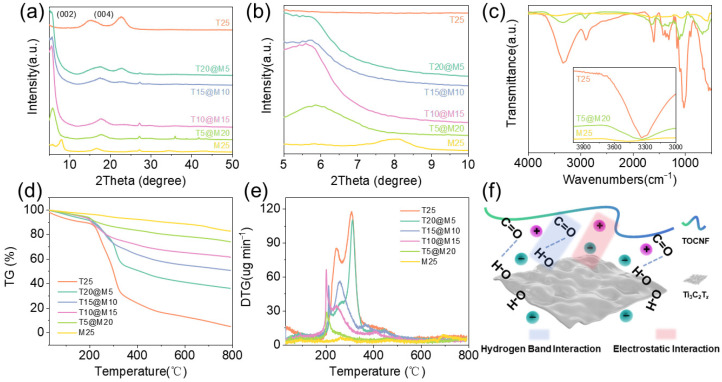
(**a**) XRD analysis results; (**b**) Magnified low-angle XRD patterns; (**c**) FTIR Spectroscopic Analysis; (**d**) TGA curves; (**e**) DTG curves; (**f**) Schematic illustration of the microscopic mechanism of the TOCNFs@Ti_3_C_2_T*_x_* hybrid film.

To elucidate the crystallographic structure and the structural evolution of the hybrid films, XRD analysis was performed (Figure 4a). As illustrated in the full-spectrum XRD patterns, T25 films exhibits broad characteristic diffraction peaks at approximately 16° and 22.5°, corresponding to the typical cellulose I crystal structure. M25 films display a sharp, intense (002) diffraction peak at around 8.0°, indicating a highly ordered layered configuration. For the TOCNFs@Ti_3_C_2_T*_x_* hybrid films, the characteristic peaks of both TOCNFs and MXene coexist, confirming the successful physical integration of the two components without disrupting their intrinsic crystal phases. Notably, the magnified low-angle XRD patterns (Figure 4b) reveal a continuous leftward shift of the MXene peak (002) upon the incorporation of TOCNFs. For the optimized T5@M20 film, the peak (002) shifts significantly to a lower angle of roughly 5.8°. The peak shift corresponds to a substantial expansion of the interlayer spacing of the MXene sheets. This crystallographic evolution provides compelling evidence that the 1D high aspect ratio TOCNFs are effectively intercalated into the 2D MXene interlayers. This intercalation successfully suppresses the severe self-restacking of MXene nanosheets driven by intrinsic van der Waals forces. Furthermore, the retention of a well-defined (002) peak profile in the highly loaded hybrid films demonstrates that the vacuum filtration coupled with hot-pressing densification effectively eliminates structural disorder, constructing a dense and highly ordered nacre-inspired layered architecture.

To further elucidate the interfacial chemical interactions during the hybridization process, FTIR was conducted on the optimized T5@M20 hybrid film, with T25 and M25 serving as control groups. In the FTIR spectrum of T25 (Figure 4c), a broad and strong absorption peak at around 3350 cm^−1^ is assigned to the stretching vibration of hydroxyl (-OH) groups on the cellulose molecular chains, while the absorption peaks at 1600 cm^−1^ and 1050 cm^−1^ correspond to the characteristic vibrations of carboxylate (-COO^−^) groups and the cellulose C-O skeleton, respectively. For M25 film, a distinct Ti-O lattice vibration peak appears in the low-wavenumber fingerprint region at 550 cm^−1^, verifying the abundant oxygen-containing functional groups on its surface. With respect to the T5@M20 hybrid film, the -OH stretching vibration peak presents a notable red-shift to lower wavenumbers in comparison with the characteristic peak at 3350 cm^−1^ for T25 films. This phenomenon confirms that a robust intermolecular hydrogen-bonding network is formed between the hydroxyl/carboxyl groups on TOCNFs chains and the terminal functional groups on the MXene surface. Furthermore, the retention of the Ti-O characteristic peak near 700 cm^−1^ and the slight variation in the peak shape of the -COO^−^ group in the hybrid film further evidence the successful hybridization of all components and the chemical interactions established between them.

To evaluate the thermal stability of the films and verify the component contents within the composites, TGA was conducted on the samples under a nitrogen atmosphere (Figure 4d,e). The T25 film displays thermal decomposition characteristics typical of cellulose-based bio-polymers, with the main weight loss occurring between 200 and 350 °C, which corresponds to the cleavage of glycosidic bonds in the cellulose chains and the dehydration carbonization of hydroxyl groups. At 800 °C, the residual mass is approximately 17 wt.%. In contrast, the M25 film exhibits excellent thermal stability within the tested range. Apart from the evaporation of a small amount of adsorbed water at low temperatures, no significant thermal degradation occurs, leaving a residual mass of roughly 88 wt.% at 800 °C. The incorporation of MXene into the TOCNFs film significantly improves its thermal stability. Taking the T5@M20 sample as an example, its thermal decomposition curve exhibits a distinct delayed effect, with a final residual mass of approximately 76 wt.% at 800 °C. According to the feeding mass ratio of MXene to TOCNFs of 4:1 and the residual rate of each component, the theoretical residual value of the sample is calculated to be 73.8 wt.%. This result confirms that the vacuum filtration method can precisely control the component ratio of the TOCNFs@Ti_3_C_2_T*_x_* hybrid films, and no significant mass loss or phase separation occurs during the hybridization process. The slightly higher final residual mass than the theoretical value may be ascribed to the interfacial interaction between TOCNFs and MXene, which promotes the formation of a more stable carbon layer [19,29].

As schematically illustrated in Figure 4f, MXene nanosheets act as the rigid skeleton and continuous conductive pathways in the hybrid system, while TOCNFs function as efficient physical spacers and binders between the MXene lamellae. The construction of this unique structure is dominated by the hydrogen-bonding network, which effectively eliminates the interlayer voids between MXene nanosheets. The high-aspect-ratio TOCNFs intercalated into the MXene interlayers can effectively suppress the intrinsic self-restacking of MXene nanosheets [30]. Meanwhile, as a stress transfer medium, TOCNFs enable efficient dissipation of external loads, which endows the hybrid films with significantly enhanced mechanical flexibility and structural stability [31,32].

### 3.2. Mechanical Properties of TOCNFs@Ti_3_C_2_T_x_ Hybrid Films

**Figure 5:**
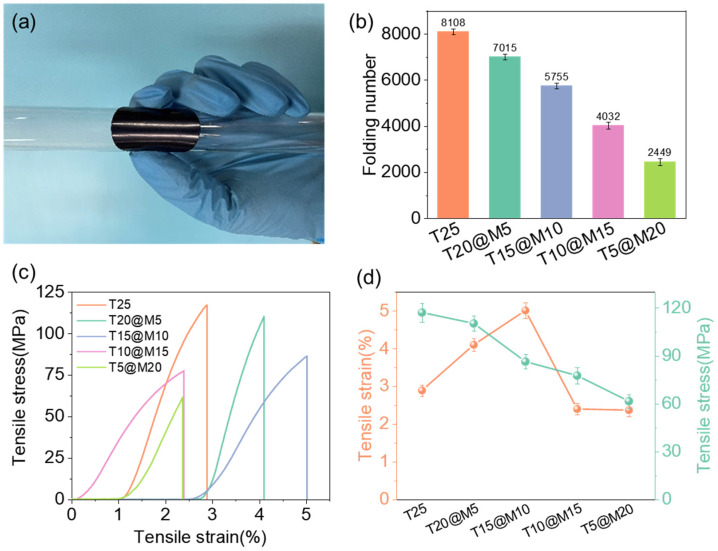
(**a**) Digital photo of T5@M20; (**b**) Folding test; (**c**) Tensile Stress–Strain curves.; (**d**) Calculated mechanical property curves.

For EMI shielding applications, the macroscopic flexibility and dynamic fatigue resistance of materials are of critical importance. M25 films are limited by weak interlayer van der Waals forces and the 2D rigid structure that is prone to stress concentration, making them difficult to withstand complex macroscopic deformations [30,33]. As shown in Figure 5a, taking the T20@M5 hybrid film as an example, the film can be attached to the surface of a test tube with a small radius of curvature, and no macroscopic cracks or structural damage are observed after severe bending. As shown in Figure 5b and Table 1, folding tests were performed on the hybrid films with different mass ratios to evaluate their dynamic fatigue resistance. The T25 film fractured after withstanding 8108 folding cycles due to its inherent polymer characteristics. The folding endurance of the films exhibits a downward trend with increasing Ti_3_C_2_T*_x_* content. Among them, the T5@M20 film fractured after 2449 folding cycles despite its extremely high MXene content, and still maintains excellent dynamic flexibility. TOCNFs play a vital role between MXene lamellae: during repeated bending of the film, the flexible TOCNFs fiber network can effectively absorb and dissipate locally concentrated stress, and retard the initiation and propagation of cracks at the edges of the rigid MXene nanosheets [34].

Tensile tests (Figure 5c) demonstrate a clear transition from brittle to ductile fracture upon TOCNFs incorporation, attributed to the high-density interfacial hydrogen-bonding network that enables nacre-inspired energy dissipation via controllable slippage. As summarized in Figure 5d and Table 1, tensile strength exhibits a downward trend with increasing MXene content, peaking at 117.15 ± 5.97 MPa for T25. Conversely, the elongation at break follows an inverted U-shaped trend, reaching its maximum of 5.01 ± 0.21% at the T15@M10 ratio. This optimal performance at T15@M10 suggests that TOCNFs effectively fill microscopic voids and facilitate efficient stress transfer, whereas excessive or insufficient loading compromises the balance between skeletal rigidity and interfacial adhesion [35,36,37].

### 3.3. Conductivity and EMI Shielding Performance of TOCNFs@Ti_3_C_2_T_x_ Hybrid Films

**Figure 6:**
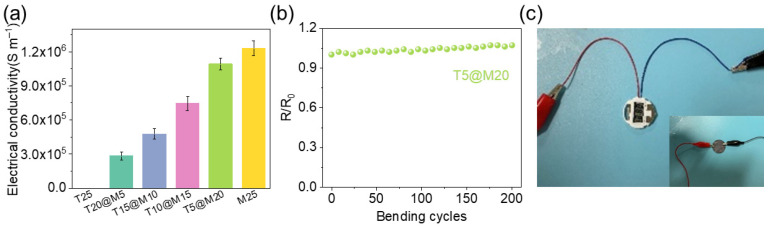
(**a**) Conductivity measurement results; (**b**) Relative resistance with the number of bending cycles; (**c**) Integrating T5@M20 into a small digital electronic display.

From a comprehensive consideration of structural reliability and practical application scenarios, in-depth analysis of the electrical properties of the hybrid films with different component mass ratios is required to achieve the optimal balance between the toughening effect and electrical properties. The characterization results of the EMI shielding performance of the hybrid films in the X-band frequency range (8.2–12.4 GHz) are summarized in Table 2. Figure 6a presents the electrical conductivity measurement results of the hybrid films with various mass ratios. The T25 film has an electrical conductivity close to 0, serving as an insulating material. The T20@M5 hybrid film achieves an electrical conductivity of 2.81 × 10^5^ ± 3.50 × 10^4^ S m^−1^, realizing the essential transition from an insulator to a conductor. This abrupt transition indicates that the percolation threshold of the composite is significantly lower than 20 wt.%. Such a low percolation threshold can be attributed to the ultrahigh electrical conductivity MXene nanosheets and the highly densified layered architecture facilitated by hot-pressing, which enable the rapid formation of a continuous conductive network even at a relatively low filler loading. With the increase in the mass proportion of Ti_3_C_2_T*_x_* in the hybrid system, the electrical conductivity of the hybrid films exhibits a monotonic increasing trend and shows a strong positive correlation with the Ti_3_C_2_T*_x_* content. The T5@M20 hybrid film reaches an electrical conductivity of 1.09 × 10^6^ ± 5.06 × 10^4^ S m^−1^. The high mass fraction of Ti_3_C_2_T*_x_* forms a denser and more continuous layered conductive framework, which reduces the interfacial contact resistance during electron transport. The electrical conductivity of the T5@M20 hybrid film reaches 88.6% of M25 film, indicating that the introduction of TOCNFs retains the high intrinsic electrical conductivity of MXene while enhancing the mechanical properties of the film. A dynamic bending cycle test was performed on the T5@M20 hybrid film (Figure 6b). During 200 consecutive bending cycles, the relative resistance ratio R/R_0_ of the film remains stable at around 1.0 without obvious fluctuations, demonstrating excellent bending fatigue resistance and electrical reliability. The T5@M20 hybrid film was integrated into a practical circuit as a conductive component (Figure 6c). As depicted, the film is securely connected via alligator clips and successfully powers a small digital electronic display. This practical demonstration directly confirms the robust macroscopic conductivity of the hybrid film, highlighting its immense potential for real-world applications in flexible electronics and integrated circuits.

**Figure 7:**
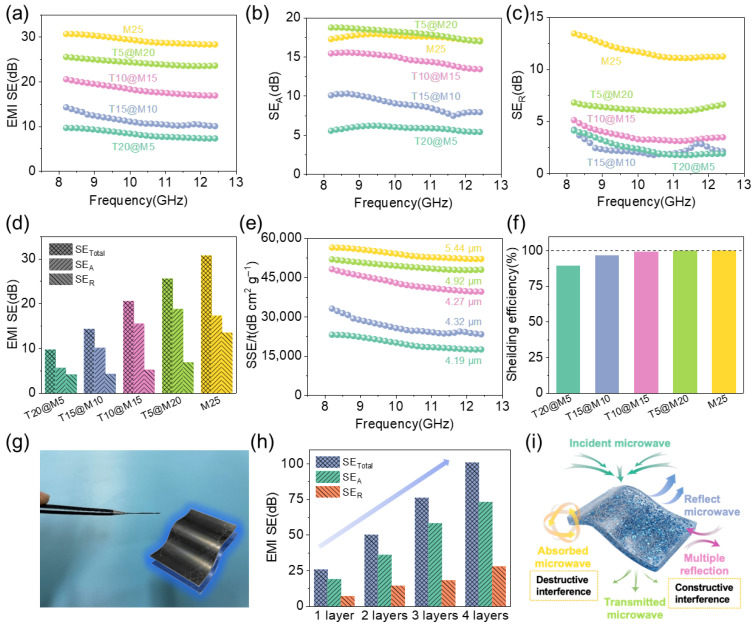
(**a**) EMI SE of the hybrid films; (**b**) SE_A_ of the hybrid films; (**c**) SE_R_ of the hybrid films; (**d**) EMI SE, SE_A_ and SE_R_ of hybrid films at 8.2 GHz; (**e**) SSE/t of the hybrid films; (**f**) Shielding efficiency of the hybrid films; (**g**) Photograph of two laminated T5@M20 layers; (**h**) EMI SE, SE_A_ and SE_R_ of the T5@M20 films with different laminated layers; (**i**) Schematic illustration of the EMI shielding mechanism of the hybrid films.

The test results of the SE_Total_ of the hybrid films (Figure 7a) show a strong correlation with the electrical conductivity measurement results. The T25 film has an electrical conductivity close to 0 and thus exhibits no EMI shielding performance. With the increase in Ti_3_C_2_T*_x_* content, the SE_Total_ of the hybrid films presents a continuous increasing trend. The T20@M5 sample has an SE_Total_ of 9.70 dB, which can only shield 89.28% of incident electromagnetic waves. In contrast, the T5@M20 sample achieves an SE_Total_ of 25.55 dB, with the shielding efficiency increased to 99.72%, which is slightly lower than 99.91% of the M25 film (Table 2). To further clarify the EMI shielding mechanism of the hybrid films, the SE_Total_ was decomposed into absorption shielding effectiveness (SE_A_) and reflection shielding effectiveness (SE_R_). Both the Ti_3_C_2_T*_x_* and TOCNFs used in this work are typical non-magnetic materials, whose magnetic loss in the X-band (8.2–12.4 GHz) is negligible. Thus, the electromagnetic absorption of the film is entirely derived from dielectric loss, including dominant conductive loss and supplementary polarization relaxation loss. The ultrahigh conductivity of the optimized T5@M20 film brings strong conductive loss via Joule heat dissipation, and the conductivity trend of all samples is highly consistent with the SE_A_ variation, verifying its decisive role in absorption. The polarization relaxation loss induced by the hybrid films further enhances the dielectric loss and electromagnetic absorption capacity. The FTIR results confirm that a dense hydrogen-bonded network is formed between TOCNFs and MXene, constructing abundant heterogeneous interfaces between the two phases. The XRD and SEM results verify that TOCNFs are successfully intercalated into the MXene interlayers, inhibiting the self-restacking of MXene and forming a highly ordered nacre-like layered structure. In an alternating electromagnetic field, charge accumulation occurs at these heterogeneous interfaces, inducing significant interfacial polarization and corresponding relaxation process, which dissipates electromagnetic energy into heat [38]. The abundant terminal functional groups on the MXene surface, as well as the hydroxyl and carboxyl groups on the TOCNFs molecular chains, act as massive dipole centers. These dipoles undergo repeated orientation and relaxation in the alternating electromagnetic field, bringing additional dipole polarization loss to further improve the absorption capacity of the material. Meanwhile, the introduction of TOCNFs optimizes the impedance matching of the films (Figure 7i), allowing more incident electromagnetic waves to enter and be attenuated by the synergistic dual losses. By comparing Figure 7b–d,f, it is found that the SE_A_ of all hybrid film samples is significantly higher than the SE_R_, indicating that the attenuation of incident electromagnetic waves by the TOCNFs@Ti_3_C_2_T*_x_* hybrid films is dominated by absorption loss [39].

Since evaluating absolute shielding effectiveness without accounting for material thickness is less meaningful for practical applications, SSE/t was calculated to accurately benchmark the hybrid films (Figure 7e). The T20@M5 sample achieves an SSE/t of 23,141.43 dB cm^2^ g^−1^. With the increase in Ti_3_C_2_T*_x_* content, the SSE/t of the T5@M20 sample reaches 51,934.72 dB cm^2^ g^−1^, which is close to 56,426.95 dB cm^2^ g^−1^ of the M25 film. Meanwhile, it has an ultrathin thickness of only 4.92 ± 0.28 μm, presenting a prominent advantage of ultra-thinness. To further boost the absolute shielding performance and evaluate the practical tunability, a lamination strategy was employed. A digital photograph (Figure 7g) displays the successful lamination of two flexible T5@M20 layers, demonstrating their excellent conformability and structural integrity. Figure 7h and Table 3 demonstrate the linear correlation between the SE_Total_ of the T5@M20 film and the number of laminated layers. The single-layer T5@M20 film has an SE_Total_ of 25.55 dB, with an electromagnetic wave shielding efficiency of 99.72%, which meets the basic application threshold for EMI shielding materials. When laminated into 4 layers, the SE_Total_ of the film reaches 100.85 dB, achieving near-complete attenuation of incident electromagnetic waves in the X-band. Linear fitting results show that the correlation coefficient R^2^ between the number of laminated layers and the SE_Total_ of the film is 0.9998, and the shielding effectiveness increases by approximately 25 dB for each additional layer of T5g@M20. For the T5@M20 sample, its SE_A_ is 18.74 dB, accounting for 73.35% of the SE_Total_, while the SE_R_ is only 6.81 dB, accounting for 26.65% of the SE_Total_. Among them, the M25 film has an SE_R_ of 13.43 dB, which is higher than that of all hybrid film samples, but its SE_A_ is 17.26 dB, slightly lower than that of the T5@M20 sample. This indicates that the introduction of TOCNFs does not weaken the electromagnetic wave absorption capacity of the material; on the contrary, it enhances the absorption shielding effectiveness of the material by constructing a multilayered structure and abundant heterogeneous interfaces [40,41]. The SE_A_ of the hybrid films shows a substantial increasing trend with the increase in Ti_3_C_2_T*_x_* content, rising from 5.56 dB for T20@M5 to 18.74 dB for T5@M20, with an increase rate of 236.7%. In comparison, the increase rate of SE_R_ is relatively moderate, at only 64.9%.

This phenomenon can be explained from the perspective of the interaction between electromagnetic waves and materials (Figure 7i). A large number of heterogeneous interfaces formed between TOCNFs and Ti_3_C_2_T*_x_* can induce interfacial polarization and dipole polarization in an alternating electromagnetic field, resulting in polarization relaxation loss, which converts incident electromagnetic energy into thermal energy for dissipation. In addition, the nacre-inspired layered structure of the hybrid films enables multiple reflections and scattering of electromagnetic waves entering the interior of the material between the MXene nanosheets, which prolongs the transmission path of electromagnetic waves and further improves the efficiency of SE_A_. In contrast, SE_R_ is mainly determined by the impedance mismatch between the free carriers on the material surface and the incident electromagnetic waves. Although the increase in Ti_3_C_2_T*_x_* content raises the free electron density on the material surface, the introduction of TOCNFs regulates the surface impedance of the material to a certain extent; thus, no significant increase synchronized with SE_A_ is observed for SE_R_ [39,42,43].

Furthermore, the variation in shielding effectiveness during the lamination process reveals the critical effect of thickness on impedance matching. As the number of laminated layers increases, the augmented overall thickness enriches the conductive network, decreasing the input impedance of the material. This exacerbates the impedance mismatch between the hybrid films and the free space (~377 Ω), resulting in stronger surface reflection. This is quantitatively evidenced by the significant increase in SE_R_ from 6.81 dB to 27.69 dB (Table 3). Nevertheless, the presence of insulating TOCNFs effectively regulates the surface impedance, preventing extreme impedance mismatch and ensuring that absorption remains the dominant shielding mechanism even at elevated thicknesses.

### 3.4. Applications of TOCNFs@Ti_3_C_2_T_x_ Hybrid Films

**Figure 8:**
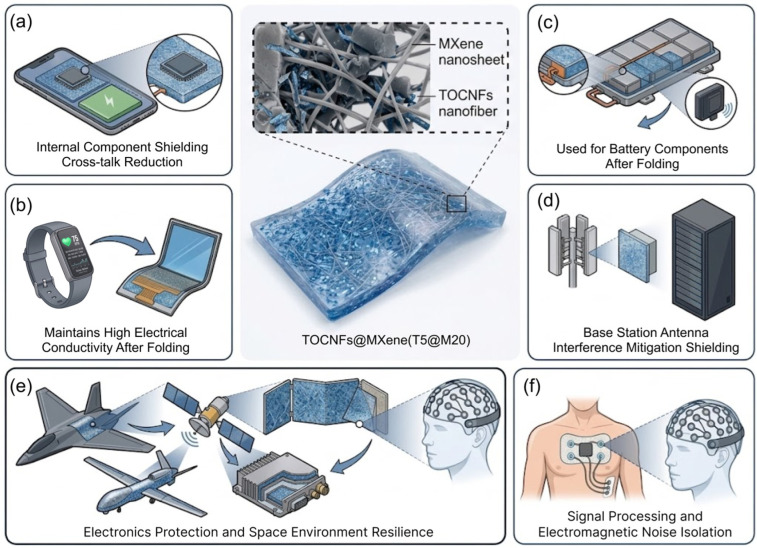
(**a**) Consumer electronics; (**b**) Flexible electronics; (**c**) EV and automotive electronics; (**d**) 5G base station data centers; (**e**) Aerospace and defense; (**f**) Medical electronics applications of TOCNFs@Ti_3_C_2_T*_x_* hybrid films. Original illustrations created via Rhino 8, Photoshop 2025, Figma, and digital drawing.

In this work, we fabricated TOCNFs@Ti_3_C_2_T_x_ hybrid films with a nacre-inspired layered structure. The optimized T5@M20 hybrid film integrates ultrathin and lightweight characteristics, mechanical flexibility, dynamic service stability, and excellent SE_Total_, breaking through the application limitations of traditional shielding materials. As shown in Figure 8, it exhibits critical engineering application value and industrialization prospects in six core fields.

As depicted in Figure 8a, in the field of flexible wearable electronics, the film is compatible with devices including smart watches, electronic skins, and flexible health monitoring equipment. It combines high-efficiency electromagnetic protection and photothermal therapy functions, satisfying the stringent requirements for long-term dynamic service of wearable devices. In the consumer electronics field, the absorption-dominated shielding mechanism of the film can effectively avoid secondary electromagnetic pollution, and its ultrahigh thickness-normalized specific shielding effectiveness is perfectly suitable for the ultrathin and lightweight design of devices such as smartphones and notebook computers (Figure 8b). In the field of new energy vehicles and automotive electronics, the film maintains stable performance under vibration conditions, and its SE_Total_ can be linearly regulated via a lamination strategy. It is applicable to the electromagnetic protection of vehicle-mounted radars, battery management systems, and electronic control units of smart cockpits, while its lightweight feature contributes to the improvement of the cruising range of the whole vehicle (Figure 8c).

The film exhibits stable and efficient shielding performance in the X-band frequency range (8.2–12.4 GHz), which can meet the protection requirements of radio frequency modules of 5G base stations and high-density server clusters in data centers. Meanwhile, it greatly reduces the weight of the shielding structure and facilitates engineering deployment (Figure 8d). In the aerospace and national defense military industry fields, its ultrahigh SSE/t can significantly reduce the dead weight of equipment, and it presents outstanding structural stability under extreme environments, enabling full-dimensional electromagnetic protection for avionics systems and guidance systems (Figure 8e). As shown in Figure 8f, benefiting from the excellent biocompatibility of TOCNFs, the film can be applied to the electromagnetic protection of precision medical equipment and implantable medical devices in the medical electronics field, ensuring the detection accuracy and operational safety of the equipment.

The fabrication strategy proposed in this study is environmentally friendly, facile in process, and suitable for large-scale production. It provides a new perspective for the structural design and application expansion of next-generation high-performance flexible EMI shielding materials, and possesses important theoretical and practical significance for promoting the industrial application of TOCNFs@Ti_3_C_2_T*_x_*-based composites.

## 4. Conclusions

In this study, TOCNFs@Ti_3_C_2_T*_x_* hybrid films with a nacre-inspired layered structure were fabricated. The optimized T5@M20 film integrates ultrathin and lightweight characteristics, mechanical flexibility, dynamic service stability, and excellent EMI shielding performance, exhibiting critical engineering application value and industrialization prospects in six core fields (Figure 8). In the fields of flexible wearable electronics and consumer electronics (Figure 8a,b), the film can satisfy the stringent requirements for long-term dynamic service. Its absorption-dominated shielding mechanism effectively avoids secondary electromagnetic pollution, and its SSE/t is perfectly suitable for ultrathin and lightweight design. In the fields of new energy vehicles and communications (Figure 8c,d), its shielding effectiveness can be linearly regulated via a lamination strategy, and its lightweight feature contributes to improvement of the cruising range of vehicles. Furthermore, its stable and efficient shielding performance in the X-band frequency range can significantly reduce the weight of shielding structures for base stations and data centers. In the aerospace and national defense military industry fields (Figure 8e), the film presents outstanding structural stability, significantly reducing the dead weight of equipment. The film can also provide safe and reliable electromagnetic protection for precision and implantable medical devices in the medical electronics field (Figure 8f). In summary, the green, facile, and easy-to-scale fabrication strategy proposed in this study provides a new perspective for the structural design and application expansion of next-generation high-performance flexible EMI shielding materials.

## Figures and Tables

**Table 1:** The mechanical properties of the TOCNFs@Ti_3_C_2_T*_x_* hybrid films with different contents.

Sample	Folding Number	Tensile Strain (%)	Tensile Strength (MPa)
T25	8108±119	2.89±0.15	117.15±5.97
T20@M5	7015±117	4.10±0.16	110.33±4.65
T15@M10	5755±129	5.01±0.21	86.42±4.55
T10@M15	4032±142	2.40±0.15	77.61±5.12
T5@M20	2449±152	2.37±0.17	61.60±4.38
M25	Cannot be clamped		

**Table 2:** Properties of the TOCNFs@Ti_3_C_2_T*_x_* hybrid films with different contents.

Sample	Thickness (μm)	Conductivity (s m^−1^)	SE_Total_ (dB)	SE_A_ (dB)	SE_R_ (dB)	SE_A_/SE_Total_ (%)	SSE/t (dB cm^2^ g^−1^)	Theoretical Shielding Efficiency (%)
T25	4.11 ± 0.21	1.34 × 10^−4^ ± 3 × 10^−6^	/	/	/	/	/	/
T20@M5	4.19 ± 0.23	2.81 × 10^5^ ± 3.50 × 10^4^	9.70	5.56	4.13	57.32	23,141.43	89.28
T15@M10	4.32 ± 0.29	4.76 × 10^5^ ± 4.63 × 10^4^	14.30	10.08	4.22	70.49	33,110.02	96.29
T10@M15	4.27 ± 0.31	7.43 × 10^5^ ± 6.16 × 10^4^	20.57	15.44	5.13	75.06	48,165.88	99.12
T5@M20	4.92 ± 0.28	1.09 × 10^6^ ± 5.06 × 10^4^	25.55	18.74	6.81	73.35	51,934.72	99.72
M25	5.44 ± 0.24	1.23 × 10^6^ ± 6.41 × 10^4^	30.70	17.26	13.43	56.22	56,426.95	99.91

**Table 3:** Electromagnetic shielding properties of T5@M20 hybrid films with different lamination layers.

Number of Laminated Layers	SE_Total_ (dB)	SE_A_ (dB)	SE_R_ (dB)	SE_A_/SE_Total_ (%)	Theoretical Shielding Efficiency (%)
1	25.55	18.74	6.81	73.35	99.72
2	50.18	35.87	14.31	71.48	99.999
3	76.00	58.12	17.88	76.47	99.9999975
4	100.85	73.16	27.69	72.54	99.999999992

## Data Availability

The raw data supporting the conclusions of this article will be made available by the authors on request.

## Abbreviations

The following abbreviations are used in this manuscript:

EMI	Electromagnetic Interference
EMI SE	Electromagnetic Interference Shielding Effectiveness
CNTs	Carbon Nanotubes
Ti_3_C_2_T*_x_*	2D Titanium Carbide MXene
TOCNFs	TEMPO-oxidized Cellulose Nanofibrils
TEMPO	2,2,6,6-Tetramethylpiperidine-1-oxyl
MXene	2D Transition Metal Carbides and Nitrides
DI	Deionized Water
SEM	Scanning Electron Microscopy
FTIR	Fourier Transform Infrared Spectroscopy
TEM	Transmission Electron Microscopy
XRD	X-ray Diffraction
rGO	Reduced Graphene Oxide
MAX	Ternary Layered Transition Metal Carbides/Nitrides (MAX phase)
AFM	Atomic Force Microscopy
TGA	Thermogravimetric Analysis
SETotal	Total Electromagnetic Shielding Effectiveness
SEA	Absorption Shielding Effectiveness
SER	Reflection Shielding Effectiveness
